# Congenital and Neonatal Skin Disorders: Histopathological Diagnosis and Syndromic Associations

**DOI:** 10.7759/cureus.90102

**Published:** 2025-08-14

**Authors:** Hussein Qasim, Mohammad Abu Shugaer, Karis Khattab, Matteo Luigi Giuseppe Leoni, Giustino Varrassi

**Affiliations:** 1 Department of Pathology and Laboratory Medicine, Jordan University of Science and Technology, Irbid, JOR; 2 Faculty of Medicine, Yarmouk University, Irbid, JOR; 3 Department of Medical and Surgical Sciences and Translational Medicine, Sapienza University, Rome, ITA; 4 Department of Pain Medicine, Fondazione Paolo Procacci, Rome, ITA

**Keywords:** congenital skin disorders, genodermatoses, neonatal dermatology, pediatric dermatopathology, syndromic skin disorders

## Abstract

Congenital and neonatal skin disorders encompass a broad spectrum of conditions ranging from transient benign rashes to severe genodermatoses and multisystem syndromes. Accurate diagnosis in the newborn period is critical, as cutaneous findings may be the earliest clue to underlying genetic, infectious, or systemic diseases. A comprehensive literature review was performed, drawing from up-to-date peer-reviewed studies, landmark dermatology and pathology reviews, and authoritative texts. Key information on clinical presentation, histology, and syndromic associations was extracted and synthesized. Congenital skin disorders often reflect disruptions in embryologic development or genetic mutations in skin structural proteins. Histopathological examination remains a cornerstone of diagnosis, revealing characteristic patterns (e.g., level of blister cleavage in epidermolysis bullosa, epidermolytic hyperkeratosis in ichthyoses, and eosinophilic spongiosis in incontinentia pigmenti). Many disorders form part of broader syndromes with distinctive cutaneous and systemic features. Recent advances in immunofluorescence mapping and genetic testing have improved diagnostic precision, while emerging therapies (e.g., gene therapy) hold promise for conditions previously managed only supportively. The care of neonates with skin disorders requires a multidisciplinary approach combining clinical evaluation, histopathology, and genetic insights. Early recognition of telltale skin findings and their syndromic context can guide timely interventions, genetic counseling, and anticipatory management of associated complications. Future developments in molecular diagnostics and targeted therapies are poised to further enhance outcomes in this vulnerable patient population.

## Introduction and background

Neonatal skin disorders are a significant concern in perinatal medicine, as the skin is both an indicator of infant health and a potential source of morbidity [1]. The neonatal period (first four weeks of life) is marked by a variety of cutaneous findings; over 90% of newborns exhibit some dermatologic manifestation, most of which are benign or transient [2]. However, a subset of lesions present at birth or shortly after can signal underlying congenital disorders or severe systemic disease [3]. These range from minor birthmarks to life-threatening genodermatoses (inherited skin disorders), neonatal infections, and markers of complex syndromes. Early identification and accurate diagnosis of such conditions are critical, as they may necessitate urgent management or prompt evaluation for internal involvement [3].

A newborn’s skin has unique characteristics that can amplify dermatologic issues [1]. It is thinner with an immature barrier function, especially in premature infants [1]. This can make neonates more susceptible to infections, permeability issues, and exaggerated responses to environmental stresses [4]. At the same time, distinctive physiological phenomena occur (e.g., transient erythema, desquamation, and vernix caseosa), which must be distinguished from pathological lesions [5].

Thus, clinicians must differentiate normal neonatal skin changes from pathological eruptions or malformations. Equally important, many congenital skin disorders have specific histopathological hallmarks that, when recognized, can confirm a clinical impression or even reveal a diagnosis not evident on examination [6]. Histopathology plays a central role in diagnosing congenital skin disorders, often in conjunction with clinical and genetic data [7]. A skin biopsy in a neonate can demonstrate features such as the level of epidermal separation in a blistering disease, patterns of hyperkeratosis in an ichthyosis, or inflammatory cell types in an eruptive rash [8]. Correlating these microscopic findings with the patient’s presentation allows for precise classification and guides further testing (such as immunofluorescence mapping or genetic analysis) [8]. Moreover, many congenital dermatoses are part of syndromic associations [9]. For example, neurocutaneous syndromes where skin lesions accompany neurological abnormalities, or metabolic disorders with cutaneous clues [10]. Recognizing these patterns enables early multidisciplinary care (e.g., neurologic or metabolic workups) and family counseling. This review highlights congenital and neonatal skin disorders with a focus on histopathology and syndromic links. It outlines skin development, classifies disorders by cause and appearance, and summarizes key diagnostic features. Common conditions and their associations with systemic syndromes are reviewed alongside diagnostic tools and current challenges. The aim is to aid early, accurate diagnosis through clinical, pathological, and genetic integration.

## Review

Methods

This narrative review is based on a comprehensive literature search conducted in PubMed, Scopus, and Google Scholar using combinations of keywords and MeSH terms such as “congenital skin disorder,” “neonatal dermatology,” “histopathology,” and “syndromic associations.” Literature published between January 2000 and May 2025 was considered, with emphasis on studies from the last five years. Additional references were drawn from authoritative dermatology and pathology textbooks. Inclusion criteria were English-language articles describing clinical presentation, histopathological features, or syndromic associations of congenital or neonatal skin disorders. Case reports were included if they illustrated rare or diagnostically significant conditions. No formal bias assessment was performed due to the descriptive and educational nature of the review. No statistical or quantitative synthesis (e.g., meta-analysis) was performed, as this work is a descriptive, narrative review.

Embryology and skin development

The development of human skin begins early in embryogenesis and continues through fetal life, with structural and functional maturation extending into the neonatal period [1]. The epidermis originates from the surface ectoderm, which by the fourth week of gestation begins to proliferate into a multilayered structure [11]. As gestation progresses, keratinocytes undergo differentiation, and by the end of the first trimester, the basic architecture of the epidermis, including the basal, spinous, granular, and cornified layers, is established [11]. Concurrently, the dermis arises from mesenchymal tissue derived from the mesoderm and neural crest [12]. Dermal fibroblasts begin to organize into connective tissue structures, while blood vessels and other mesodermal elements support the developing skin [13]. Appendageal structures such as hair follicles, sebaceous glands, and sweat glands start to form between the ninth and 12^th^ weeks and continue their maturation until mid-gestation [14]. Melanocytes, derived from the neural crest, migrate into the basal layer of the epidermis during the first trimester and begin producing melanin around the 14^th^ to 16^th^ week [15]. The maturation of the stratum corneum, critical for barrier function, occurs primarily in the third trimester [16]. This developmental milestone is particularly relevant in premature neonates, whose skin often lacks adequate barrier function, making them more susceptible to transepidermal water loss, trauma, and infection [16]. Disruptions in these tightly regulated developmental processes can result in a wide array of congenital skin disorders [17]. For example, defective anchoring between the epidermis and dermis can lead to blistering diseases such as epidermolysis bullosa [18]. Mutations affecting epidermal differentiation may result in disorders like lamellar ichthyosis, while aberrant melanocyte migration or function is implicated in pigmentary disorders such as piebaldism and hypomelanosis of Ito [19]. A thorough understanding of skin embryology is therefore essential for interpreting neonatal dermatological findings and for identifying the underlying pathophysiological mechanisms [20].

Classification of congenital and neonatal skin disorders

Congenital and neonatal skin disorders can be classified using several frameworks, including etiology, morphology, and time of onset [1]. Such classification aids clinicians and pathologists in developing a structured approach to diagnosis and management [21]. From an etiological perspective, these conditions may arise from genetic mutations, intrauterine infections, vascular abnormalities, inflammatory responses, or metabolic derangements [22]. Genetic disorders constitute a large proportion of congenital dermatoses, with mutations affecting structural proteins, enzymes, or signaling pathways essential for skin development [23]. Infectious causes, such as intrauterine transmission of herpes simplex virus, syphilis, or cytomegalovirus, can also result in distinctive cutaneous lesions at or shortly after birth [24]. Inflammatory and immune-mediated diseases, though less common in neonates, may include presentations of neonatal lupus or congenital variants of immunodeficiency syndromes [25]. Vascular anomalies, including capillary malformations and hemangiomas, also commonly present in the neonatal period, with varied clinical implications [26]. Morphologically, neonatal skin disorders can be grouped into categories such as bullous or vesiculobullous eruptions, ichthyotic or hyperkeratotic conditions, pigmentary abnormalities, and vascular lesions [27]. Bullous lesions are characteristic of diseases like epidermolysis bullosa or staphylococcal scalded skin syndrome [28]. Hyperkeratotic conditions, including congenital ichthyoses, present with thick scaling due to disrupted epidermal turnover [29]. Pigmentary disorders may involve hypo- or hyperpigmented patches and often have neurocutaneous or genetic correlates [30]. Vascular lesions, such as port-wine stains and infantile hemangiomas, represent anomalies of blood vessel formation and growth and may be isolated or part of syndromic conditions [31]. The timing of presentation provides another helpful axis of classification [2]. True congenital dermatoses are evident at birth, while others manifest within the first month of life and are classified as neonatal [32]. Conditions like aplasia cutis congenita, congenital melanocytic nevi, and epidermolytic ichthyosis are typically visible at birth [33]. In contrast, neonatal lupus, erythema toxicum neonatorum, and transient neonatal pustular melanosis may develop days to weeks after delivery [34]. Integrating these axes, etiological, morphological, and chronological, enables a comprehensive and nuanced understanding of congenital and neonatal skin diseases [35]. This structured classification not only improves diagnostic accuracy but also facilitates identification of disorders with syndromic associations that may require broader systemic evaluation [36].

Histopathological features of common disorders

Histopathological examination plays a central role in the diagnosis of congenital and neonatal skin disorders, especially when clinical features are subtle, overlapping, or part of a broader syndromic context [37]. Many conditions display characteristic microscopic patterns that can be diagnostic or at least strongly suggestive when interpreted alongside clinical findings [38]. Epidermolysis bullosa, a group of inherited blistering disorders, is a classic example where histopathology, often supplemented by immunofluorescence mapping and electron microscopy, is essential for diagnosis [39]. Epidermolysis bullosa is classified based on the level of skin cleavage within the basal keratinocytes (epidermolysis bullosa simplex), through the lamina lucida (junctional epidermolysis bullosa), or beneath the lamina densa (dystrophic epidermolysis bullosa) [40]. Light microscopy may show intraepidermal, junctional, or subepidermal blistering, but definitive classification often requires identification of specific protein deficiencies such as keratin 14, laminin-332, or type VII collagen [41]. Ichthyoses represent another group of genetically determined disorders characterized by scaling and hyperkeratosis [42]. Histologically, lamellar ichthyosis shows compact orthokeratotic hyperkeratosis with a diminished or absent granular layer, whereas epidermolytic ichthyosis is marked by epidermal hyperplasia, cytolysis in the upper epidermis, and coarse keratohyalin granules, reflecting keratin mutations [43]. Incontinentia pigmenti, an X-linked dominant disorder affecting skin, teeth, eyes, and the central nervous system, shows a staged progression of histological findings [44]. Early vesiculobullous lesions reveal eosinophilic spongiosis and intraepidermal vesiculation, while later stages display interface dermatitis and dermal pigment incontinence [45]. These findings parallel the clinical evolution of the disorder, from inflammatory vesicles to verrucous plaques, followed by hyperpigmentation and eventual atrophic hypopigmentation [46]. Neonatal lupus erythematosus, an autoimmune condition resulting from transplacental passage of maternal anti-Ro and anti-La antibodies, typically presents with annular erythematous plaques on the face and scalp [47]. Histologically, it mimics subacute cutaneous lupus erythematosus, showing vacuolar interface dermatitis, epidermal atrophy, and a perivascular lymphocytic infiltrate with mucin deposition [48]. Langerhans cell histiocytosis (LCH), a clonal proliferation of dendritic cells, may present in neonates with crusted papules and erosions resembling seborrheic dermatitis or scabies [49]. Skin biopsy reveals an infiltrate of large, pale-staining histiocytic cells with reniform nuclei, often admixed with eosinophils [50]. Immunohistochemical staining for CD1a, S100, and Langerin (CD207) confirms the diagnosis [51]. Vascular anomalies such as infantile hemangiomas and capillary malformations may also be biopsied when the diagnosis is unclear [52]. Hemangiomas show proliferative endothelial cells forming small capillary-like vessels during the growth phase, transitioning to fibrofatty involution in later stages [53]. Capillary malformations, in contrast, exhibit ectatic dermal capillaries lined by flattened endothelium without cellular proliferation [54]. Pigmentary disorders like piebaldism and albinism typically show a reduced or absent number of melanocytes and melanin in the basal layer [55]. In some cases, Fontana-Masson staining and immunohistochemical markers like Melan-A and HMB-45 can aid in visualizing melanocytes [56]. Histopathology thus serves as a cornerstone in the diagnostic work-up of congenital and neonatal skin conditions, especially when paired with targeted immunohistochemistry and, increasingly, genetic studies (Table 1). Early biopsy and accurate interpretation can guide definitive diagnosis, inform prognosis, and prompt evaluation for associated systemic or genetic disorders.

**Table 1:** Histopathological features of selected congenital and neonatal skin disorders EM: erythroderma multiforme; LI: lamellar ichthyosis; EI: epidermolytic ichthyosis; DIF: direct immunofluorescence This table has been created by the authors based on the findings from the studies included in the review.

Disorder	Histopathological features	Diagnostic aids
Epidermolysis bullosa	Level-specific skin cleavage (intraepidermal, junctional, subepidermal)[41]	Immunofluorescence mapping, EM [57]
Ichthyosis (e.g. LI, EI)	Hyperkeratosis, variable granular layer, vacuolar degeneration (in EI) [58]	Genetic testing, keratin staining [42]
Incontinentia pigmenti	Eosinophilic spongiosis → interface dermatitis → dermal pigment incontinence [59]	Stage-dependent, biopsy timing crucial [60]
Neonatal lupus	Vacuolar interface dermatitis, epidermal atrophy, perivascular lymphocytes, mucin [61]	Anti-Ro/La antibodies, DIF [62]
Langerhans cell histiocytosis	Dermal infiltrate of reniform cells with eosinophils [63]	CD1a+, Langerin+, S100+ [64]
Hemangioma (proliferative)	Capillary lobules with plump endothelial cells [65]	GLUT1+, biopsy rarely needed [66]
Piebaldism/Albinism	Reduced or absent melanocytes and melanin in the basal layer [67]	Fontana-Masson stain, melanocyte IHC [68]

Syndromic associations

Many congenital and neonatal skin disorders serve as cutaneous markers of underlying multisystem syndromes [69]. In some cases, skin findings precede or outshine other systemic manifestations, positioning the dermatologist or neonatologist at the front line of syndrome recognition [70]. Neurocutaneous syndromes, or phakomatoses, exemplify this relationship between skin and systemic involvement [71]. Tuberous sclerosis complex presents with hypopigmented macules (“ash leaf” spots), facial angiofibromas, and shagreen patches, often evident at birth or in early infancy [72]. These skin findings are critical clues to underlying cortical tubers, cardiac rhabdomyomas, and renal angiomyolipomas [73]. Similarly, Sturge-Weber syndrome is marked by a facial port-wine stain, usually in the V1 dermatome, associated with leptomeningeal angiomatosis and glaucoma [74]. Neurofibromatosis type 1 (NF1) may not be fully expressed in the neonatal period, but the presence of multiple café-au-lait macules should prompt further surveillance for associated ocular, skeletal, and neurological features [75]. Genodermatoses with systemic features also frequently present with characteristic neonatal skin lesions [76]. For instance, Netherton syndrome, an autosomal recessive disorder caused by mutations in the SPINK5 gene, manifests with ichthyosiform erythroderma, hair shaft abnormalities, and immune dysregulation [77]. Similarly, ectodermal dysplasias, a group of inherited disorders affecting hair, teeth, nails, and sweat glands, may be suspected in neonates presenting with sparse hair, periorbital wrinkling, and dry or hypohidrotic skin [78]. Menkes disease, a disorder of copper metabolism, may be heralded by peculiar kinky hair and hypopigmentation, even before neurodegeneration becomes apparent [79]. Metabolic and storage disorders also frequently present with distinctive skin findings in neonates [80]. In Fabry disease, angiokeratomas may emerge in infancy, often before renal or cardiac involvement becomes symptomatic [81]. Other storage disorders, such as Niemann-Pick and Gaucher disease, can present with generalized skin infiltration or xanthomatous lesions due to lipid-laden macrophage accumulation [82]. In neonatal-onset ichthyosis syndromes, such as Chanarin-Dorfman syndrome, vacuolated neutrophils (Jordan’s anomaly) seen in peripheral blood smears provide a systemic clue that complements the dermatological picture [83]. Immunodeficiency syndromes may also manifest cutaneously in neonates [84]. Wiskott-Aldrich syndrome, an X-linked immunodeficiency, often presents with eczema-like dermatitis, thrombocytopenia, and recurrent infections [85]. Omenn syndrome, a variant of severe combined immunodeficiency, is characterized by erythroderma, alopecia, and eosinophilia, findings that may initially mimic seborrheic or atopic dermatitis [86]. Overall, the presence of congenital skin abnormalities should prompt clinicians to consider possible syndromic associations, particularly when findings are bilateral, segmental, widespread, or accompanied by systemic signs [87]. Table 2 presents a summary of syndromic associations with neonatal skin disorders.

**Table 2:** Syndromic associations with neonatal skin disorders This table has been created by the authors based on the findings from the studies included in the review.

Syndrome	Cutaneous manifestations	Associated systemic features
Tuberous sclerosis [88]	Hypopigmented macules, facial angiofibromas, shagreen patches	Cortical tubers, cardiac rhabdomyomas, epilepsy
Sturge-Weber syndrome [89]	Facial port-wine stain (V1 distribution)	Leptomeningeal angiomatosis, glaucoma
Neurofibromatosis type 1 [90]	Café-au-lait macules, axillary freckling	Optic glioma, skeletal dysplasia, Lisch nodules
Netherton syndrome [91]	Erythroderma, ichthyosis, hair shaft defects	Atopy, recurrent infections
Ectodermal dysplasias [78]	Sparse hair, dry/hypopigmented skin	Hypodontia, anhidrosis, nail dysplasia
Menkes disease [92]	Kinky hair, hypopigmentation	Neurodegeneration, vascular tortuosity
Wiskott-Aldrich syndrome [93]	Eczematous dermatitis	Thrombocytopenia, immune deficiency
Fabry disease [94]	Angiokeratomas (rare in neonates)	Renal, cardiac, and neurologic dysfunction

Diagnostic approach

A methodical and multidisciplinary approach is essential for diagnosing congenital and neonatal skin disorders [95]. Given the potential overlap in clinical appearance and the possibility of systemic involvement, accurate diagnosis often relies on a combination of clinical assessment, histopathological analysis, and genetic or immunologic investigations [96]. Skin biopsy remains a cornerstone of this approach, especially in conditions with ambiguous presentation or when a syndrome is suspected [97]. The decision to perform a biopsy in neonates should balance diagnostic yield with procedural risks, as their skin is thinner and more delicate than in older children or adults [98]. Punch biopsy, typically 3-4 mm in diameter, is the preferred technique due to its simplicity and minimal invasiveness [99]. In cases of suspected blistering disorders or vasculitis, incisional biopsy at the margin of an active lesion can yield more informative histopathological results [100]. Adequate handling of the tissue is crucial: fresh tissue may be required for direct immunofluorescence studies, while formalin-fixed samples are processed for routine histology [101]. Histological analysis can reveal characteristic epidermal or dermal changes, patterns of inflammation, pigmentary abnormalities, or structural protein deficiencies [102]. However, in some disorders, particularly those with subtle or nonspecific histopathologic findings, further diagnostic tools are needed [103]. Immunohistochemistry can help identify specific cellular markers, for example, CD1a and Langerin in LCH or collagen VII in dystrophic epidermolysis bullosa [104]. Specialized stains, such as Fontana-Masson for melanin or periodic acid-Schiff (PAS) for glycogen storage, may also be valuable [103]. In recent years, genetic testing has become increasingly integral to the diagnostic workflow [105]. For many congenital dermatoses, including ichthyoses, epidermolysis bullosa subtypes, and pigmentary disorders, gene panels or whole-exome sequencing can confirm diagnoses and assist with prognostication, genetic counseling, and prenatal diagnosis in future pregnancies [106]. In some cases, skin biopsy can serve as a source of DNA or aid in correlating genotype with histologic phenotype [107]. Other diagnostic adjuncts may include electron microscopy, particularly useful in blistering disorders to localize the level of skin separation, and radiographic imaging, audiology, or neuroimaging when syndromic involvement is suspected [107]. Furthermore, laboratory evaluation for autoantibodies, serum enzymes, or immune cell profiles may support diagnoses in autoimmune or metabolic conditions [108]. Ultimately, the diagnostic evaluation of congenital and neonatal skin disorders benefits from close collaboration among dermatologists, pathologists, geneticists, and pediatric subspecialists [109]. Early and accurate identification not only clarifies the skin diagnosis but also often uncovers systemic disease, enabling timely intervention and long-term care planning [110].

Challenges in dermatopathology in neonates

Interpreting skin biopsies in neonates presents several unique challenges, arising from both physiological differences in immature skin and the subtlety or overlap of histopathological features among various disorders [111]. Neonatal skin differs structurally from that of older children and adults in several ways; it is thinner, with a more compact stratum corneum, a reduced number of adnexal structures, and less mature dermal collagen [112]. These developmental characteristics can mimic or obscure pathological changes, making histological interpretation more complex. Conditions such as incontinentia pigmenti or neonatal lupus progress through different clinical and histological stages, which can make the timing of the biopsy critical [113]. Early-stage lesions may show inflammatory features that later evolve into atrophic or pigmentary changes, and biopsies taken at a single time point may yield incomplete diagnostic information [114]. This temporal variability necessitates careful clinical-pathological correlation and, when needed, follow-up biopsies [115]. In addition, many congenital dermatoses share overlapping features, such as hyperkeratosis, interface dermatitis, or dermal inflammation, that limit specificity [116]. For example, early ichthyotic conditions may resemble erythrodermic inflammatory disorders, while immunodeficiency-related dermatitis can be difficult to distinguish from atopic or seborrheic presentations histologically [117]. Reliance on histology alone in these settings may lead to inconclusive or misleading diagnoses. Furthermore, the rarity of many congenital skin disorders means that pathologists may have limited experience with their histological presentations [118]. Expertise in pediatric dermatopathology is essential, and consultation with reference centers may be necessary in difficult or ambiguous cases [119]. Another constraint is the limited size of biopsy samples in neonates, which can preclude complete evaluation or the ability to perform multiple ancillary tests on a single specimen [120]. Technical limitations can also impact diagnostic clarity. Some tissues degrade rapidly or may be mishandled during processing, especially when immunofluorescence or electron microscopy is required [121]. This underscores the need for appropriate sample triage and coordination between clinicians and laboratory personnel at the time of biopsy. Lastly, ethical and practical considerations in critically ill or preterm neonates may delay or preclude biopsy, despite clinical indications [122]. In such cases, non-invasive tools such as dermoscopy, imaging, or molecular diagnostics using blood or saliva may serve as adjuncts, though they rarely replace histological analysis entirely [123]. Despite these challenges, skin biopsy remains a vital diagnostic tool in neonates [124]. Its value can be maximized through careful technique, multidisciplinary collaboration, and integration with clinical, genetic, and laboratory findings.

Future directions

**Figure 1:**
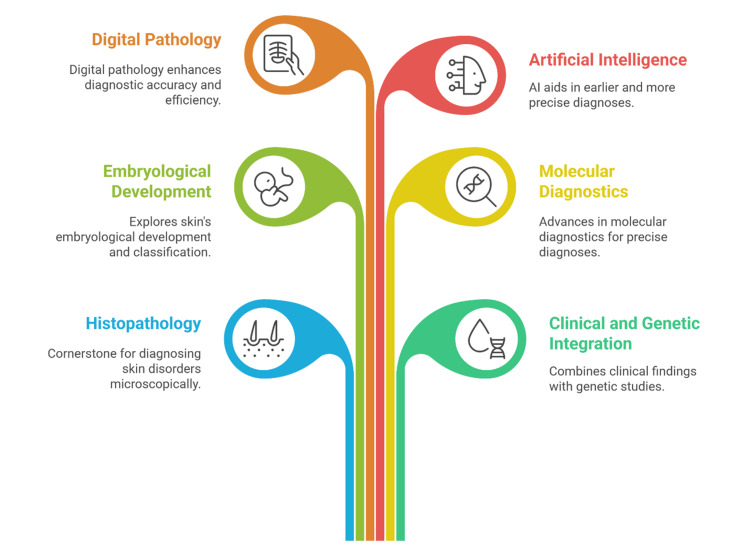
Key domains in the diagnosis and evaluation of congenital and neonatal skin disorders. This figure has been created by the authors.

The diagnostic landscape for congenital and neonatal skin disorders is rapidly evolving, driven by advances in molecular genetics, digital pathology, and integrative diagnostic approaches [125]. One of the most transformative developments has been the increasing availability and affordability of next-generation sequencing (NGS), including gene panels, whole-exome sequencing (WES), and whole-genome sequencing (WGS) [126]. These tools have enabled earlier and more precise genetic diagnoses, particularly in disorders with overlapping clinical and histological features [126]. As a result, clinicians can now move more swiftly from phenotype to genotype, even in cases where histopathology is inconclusive. Another promising frontier lies in the integration of molecular pathology with traditional histology [110]. Techniques such as immunohistochemistry, in situ hybridization, and transcriptomics are becoming more refined and accessible, allowing for more targeted diagnosis and mechanistic understanding of skin diseases [127]. For example, identifying specific protein expression patterns in blistering diseases or inflammatory pathways in genodermatoses not only improves diagnostic accuracy but also opens avenues for personalized therapy [128]. Digital pathology and artificial intelligence (AI) are also beginning to impact neonatal dermatopathology [129]. Machine learning algorithms are being trained to recognize complex histologic patterns and may eventually aid in the interpretation of rare or subtle lesions [130]. Although these tools are still in early development, they hold promise for enhancing diagnostic consistency, especially in centers lacking subspecialty expertise [131]. Looking ahead, the potential for targeted therapies based on molecular findings is growing. For instance, biologic agents and gene therapies are under investigation for some inherited disorders of keratinization and immune dysregulation [132]. Early diagnosis through integrated clinical-pathological-genetic approaches may allow neonates with severe disorders to benefit from disease-modifying interventions before irreversible damage occurs. The integration of digital pathology, artificial intelligence, molecular diagnostics, and clinical-genetic correlation is transforming the diagnostic landscape of neonatal dermatology (Figure 1). These advances support earlier, more precise, and multidisciplinary evaluation of congenital and neonatal skin disorders.

## Conclusions

Congenital and neonatal skin disorders, though often rare and diagnostically challenging, offer a unique window into a broad array of genetic, metabolic, infectious, and systemic conditions. Because many of these disorders present early and visibly on the skin, they provide a critical opportunity for prompt recognition and intervention, especially when they are part of a wider syndromic picture. Histopathological examination remains a central diagnostic tool, offering detailed insights into epidermal and dermal architecture, cellular patterns, and disease-specific markers. When combined with clinical evaluation, immunohistochemical analysis, and increasingly accessible genetic testing, histology helps establish accurate diagnoses and uncover underlying systemic associations. A thoughtful, multidisciplinary approach that includes dermatologists, pathologists, geneticists, and pediatric subspecialists is essential for optimizing outcomes. Recognizing the limitations and evolving potential of dermatopathology in neonates, clinicians must be vigilant, methodical, and collaborative in evaluating skin lesions that may signal deeper pathology. As diagnostic technologies continue to advance through molecular innovations, digital imaging, and AI, the future holds promise for earlier, more precise, and less invasive diagnosis. Ultimately, early identification and comprehensive assessment of congenital and neonatal skin disorders not only improve dermatologic care but also enable life-saving systemic evaluation and therapy.
